# Resolving Vitamin D Deficiency in the Preconception Period among High-Risk Reproductive Women: A Randomized Controlled Trial

**DOI:** 10.5812/ircmj.11175

**Published:** 2014-01-05

**Authors:** Mahshid Taheri, Azam Baheiraei, Abbas Rahimi Foroushani, Maryam Modarres

**Affiliations:** 1 Nursing and Midwifery Care Research Center, Tehran University of Medical Sciences, Tehran, IR Iran; 2 Department of Reproductive Health, Tehran University of Medical Sciences, Tehran, IR Iran; 3 Department of Epidemiology and Biostatistics, School of Public Health, Tehran University of Medical Sciences, Tehran, IR Iran; 4 Department of Midwifery, School of Nursing and Midwifery, Nursing and Midwifery Care Research Center, Tehran University of Medical Science, Tehran, IR Iran

**Keywords:** Vitamin D Deficiency, Dietary Supplements, Preconception

## Abstract

**Background:**

Although vitamin D deficiency has been linked to potential complications in reproductive women, the recommended intake dosage of this vitamin in populations with high incidence of deficiency in preconception period has not been defined.

**Objectives:**

The study investigated the effect of consuming a dosage of 2000 IU/day oral vitamin D for 105 days, on serum levels of this vitamin in reproductive women.

**Materials and Methods:**

229 women with 18-35 years old, who were confirmed to be vitamin D deficient (vitamin D < 75 nmol/L), were randomized into the intervention and control groups and after 15 weeks consumption of the supplement and placebo, their serum samples were obtained.

**Results:**

At baseline the mean serum levels of vitamin D in the control group was 23.34 ± 15.87 nmol/L and in intervention group was 25.13 ± 18.46 nmol/L, that these values didn’t have any significant difference (P = 0.43), while after intervention, significant differences between the two groups was noticed (P < 0.001). The affecting factors to achieve normal range of vitamin D in the intervention group included basal amounts of vitamin D and two underlying factors based on questionnaire data: use of oral supplements (except vitamin D and calcium) in daily life and perfect sun exposure.

**Conclusion:**

This study showed positive effect of the 2000 IU/day oral vitamin D on the serum level elevation of this vitamin in reproductive women.

## 1. Background

Vitamin D deficiency and insufficiency among veiled women have been reported to be so prevalent (1-4). The adverse effects of this deficiency are bone loss, bone turnover and myopathy in pregnant women and rickets, craniotabes, and lower bone mineral content in fetus(5); also, it has been related to the low birth weight, autoimmune disease, heart disease, cancers, cesarean section, bacterial vaginosis, preeclampsia, abortion and preterm labor (6-8). Moreover, this deficiency has been associated with low physical activity which could lead to obesity, high blood pressure and coronary artery disease (9-11).

The role of vitamin D as a modulator in the immune system (12) is very important among reproductive women. Scientists reported that the best solution for this deficiency in absence of sun exposure is consumption of vitamin D supplements (13). Although in 2010, IOM (Institute Of Medicine) recommended 600 IU/day vitamin D for 1- 70 years old persons (14), this dosage is only enough for people who have normal ranges of 25-hydroxy vitamin D3 (15, 16) and exact amounts of supplementation for population with high-risk of deficiency, in a limited time such as preconception period, is yet unclear (17, 18). Because of influential role of Vitamin D in reproductive women and their future pregnancies, reaching to normal ranges of this vitamin in a minimum time and with minimum dosage of supplementation is necessary.

## 2. Objectives

This study investigated the effect of 2000 IU/day oral vitamin D for 105 days, on serum levels of this vitamin among reproductive women in high-risk population for vitamin D deficiency.

## 3. Materials and Methods

This paper presents some findings of a major study evaluating the effect of the current intervention on the vaginal infection treatment during preconception period. The method of the whole project was similar; so the main project was registered in Iranian registry of clinical trial (IRCT201105096284N2). Between June 2011 and March 2012, using convenience sampling, eligible women aged 18 to 35 who referred to gynecology clinic of Tehran Imam-Khomeini hospital, were enrolled after signing the informed consent.

According to the study on vitamin D levels of Iranian mothers (19), the population of this study was high-risk about vitamin D deficiency; these high-risk women must had resided in the city, had stable medical condition during past six months, and didn’t have criteria such as pathological obesity, tobacco use, history of hypercalcemia, pregnancy, hepatic or renal dysfunction, malignancy or malabsorption, using medications that suppress the immunity or interfere with vitamin D metabolism. Also they should not had taken vitamin D or calcium supplements. They knew that they would be excluded from the study in case they didn’t wish to continue using drugs, become pregnant or have symptoms of sensitivity to the drug. Demographic and life style questionnaire was completed by interviewing with 237 persons and their blood samples were obtained.

**Figure 1.**
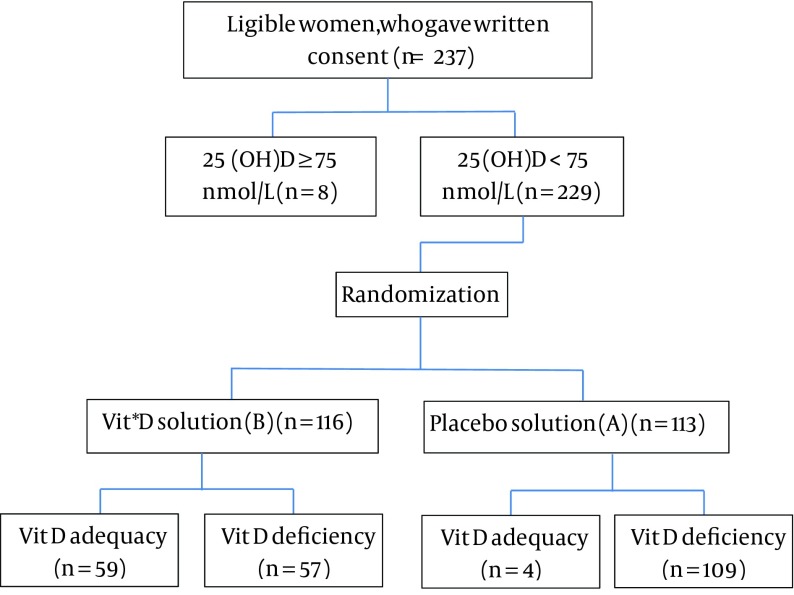
Consort Flow Diagram of Participants * vitamin

Women clot samples were transferred to the laboratory and were centrifuged at 4◦C for 15 min to separate serum. Serums were stored at -20◦C until analysis time. 25(OH)D was measured by EIA (Enzymatic ImmunoAssay) method using UK-made IDS (Immuno Diagnostic Systems) kits at the beginning of the study and after the intervention. The estimated sample size of the main project was 190 in the registered protocol; but with considering the aim of the current study, estimated sample size was increased slightly. Using online sample size estimation (available at: http://openepi.com/v37/SampleSize) and considering 90% power and 95% two-sided significance level to detect a 20% success in the experimental group, a sample size of 228 was estimated. In sampling process only 8 cases (from 237 women) had normal values of serum vitamin D (> 75nmol/L) and we removed them to homogenize the samples, so finally 229 women entered to the randomization stage (Figure 1). 

In this study, using the block randomization method, cases were randomly divided into the intervention and control groups. The list of allocation sequence was created by computer-generated randomization and based on it, each women with unique identification number was assigned to study groups. 116 women of intervention group consumed 2 drops/day of an oily vitamin D solution (equivalent to 2000 IU) and 113 women of control group received the same amounts of placebo with the similar color, smell, taste and appearance (both solutions were made by pharmacist of relevant university and were used for 105 days).

The current study was double-blind; vitamin D and placebo solutions were labeled as A and B by the pharmacist. During the study, researchers and participants didn’t have any information about real contains of these solutions. A was the placebo and B was the vitamin D solution. Participants were phone called every two weeks to ensure proper use of the drops and their cooperation. After 15 weeks, women were told to stop the usage of drops and were referred to the clinic. New blood samples were collected for laboratory examination and clot samples were evaluated by the laboratory methods used at the beginning of the study. Only 3 women were not eager to continue the study and were replaced. Results were analyzed by SPSS version 20 software using paired T test, independent T test, linear regression and chi-square tests. Relevant university ethics committee approved this study. Also at the end of the study, participants were informed about their group and their vitamin D levels.

## 4. Results

**Figure 2.**
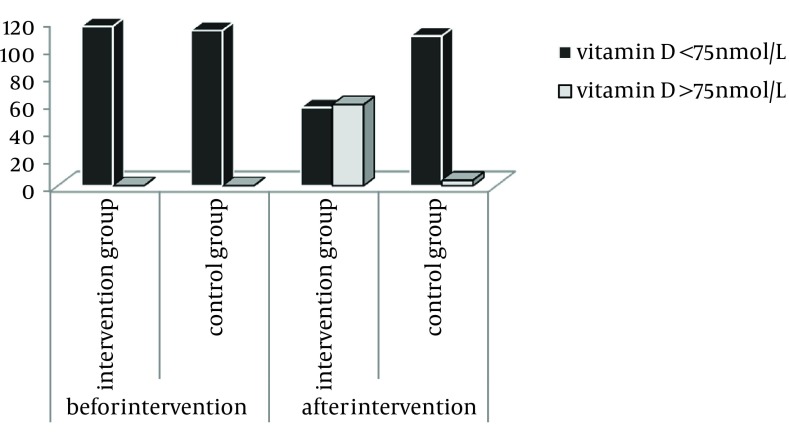
Before Intervention Serum levels of Vitamin D in Both Group Represented the Vitamin D Deficiency But at the End of Trial More Than Half of Samples in Intervention Group had Normal Status

The prevalence of vitamin D deficiency was 96.62% in reproductive women. Baseline mean of serum 25(OH) D was 23.34 ± 15.87 nmol/L in the control group and 25.13 ± 18.46 nmol/L in the intervention group without any significant difference (P = 0.43). While they were 24.74 ± 19.23 nmol/L and 74.25 ± 45.12 nmol/L after intervention, respectively (P > 0.001). There were no significant differences between the two groups with respect to demographic variables. This intervention had a positive effect on the serum levels of vitamin D (P > 0.001), even after control of confounding variables, so that the mean serum levels of vitamin D in the intervention group became 47.67 nmol/L (Standard error = 5.58) more than the control group.In the interventional group still 49.14% (n = 57) of cases had less than normal levels of vitamin D (Figure 2). 

After studying the confounding factors in this group, it was noticed that people with normal vitamin D levels after the intervention, had higher baseline mean than the others (33.06 ± 19.97 nmol/L in comparison to 16.91 ± 12.32 nmol/L); also, based on the life style history which was collected by questionnaire, routine use of oral supplements (except vitamin D and calcium which were exclusion criteria) in daily life (P = 0.03) and perfect sun exposure (P > 0.001) impacted on achieving normal serum vitamin D levels (Table 1). 

**Table 1.** Impact of Underlying Factors on Achieving Normal Levels of Vitamin D in Intervention Group

Variable	serum 25 (OH) D		
	> 75nmol/L, No (%)	< 75nmol/L, No (%)	Chi-Square
**BMI^a^**	-	-	P = 0.93
Normal	27 (50)	27 (50)	-
Abnormal	31 (51)	30 (49)	-
**Hormonal Contraceptive**	-	-	P = 0.94
Yes	7 (50)	7 (50)	-
No	51 (51)	49 (49)	-
**Economic Status**	-	-	P = 0.62
Appropriate	45 (52)	42 (48)	-
Inappropriate	13 (46)	15 (54)	-
**Second Hand Smoker**	-	-	P = 0.59
Yes	22 (48)	24 (52)	-
No	37 (53)	33 (47)	-
**Supplementation** ^**b**^ ** in Daily Life**	-	-	P = 0.03 ^c^
Yes	28 (64)	16 (36)	-
No	31 (43)	41 (57)	-
**Having Stress in Last Month**	-	-	P = 0.21
Yes	36 (47)	41 (53)	-
No	23 (59)	16 (41)	-
**Doing Regular Sport**	-	-	P = 0.08
Yes	16 (67)	8 (33)	-
No	43 (47)	49 (53)	-
**Exposing to Sunlight in Daily Life**	-	-	P < 0.001 ^c^
Perfect	18 (78)	5 (22)	-
Poor	41 (44)	52 (56)	-

^a^Abbreviations: BMI: Body Mass Index

^b^Except Vitamin D Supplements

^c^P < 0.05 is Significant

## 5. Discussion

The study reports an excessive high rate of vitamin D deficiency among reproductive women of Iran (96.62%). The prevalence of vitamin D deficiency of this study is greater than the previously stated prevalence in pregnant women, both in Iran (86%) (19) and other countries (66.7%) (20) which it can be due to the defined cut-point or the method of 25 (OH) D measurement. The study’s goal was to evaluate the impact of the higher dose of vitamin D supplementation in the short-term of pre-conception period. This study determined that the dosage of 2000 IU/day oral vitamin D is effective on serum levels of 25 (OH)D, although it failed to reach normal levels in a large number of cases (The normal levels of vitamin D for pregnant women has been reported to be 75-250 nmol/L (21)).

By statistical analysis of the intervention group, it was found that basal amounts of vitamin D, exposing to sunlight and consuming dietary supplements (except vitamin D and calcium which were exclusion criteria) in daily life were effective in reaching to normal ranges of vitamin D. The intervention by higher dosage of oral vitamin D among reproductive or pregnant women had been done by several researchers. Hollis et al. (22) after intervention in pregnant women by 2000 or 4000 IU of vitamin D/day until delivery, demonstrated that both of these dosages had significantly improved 25(OH) D in mothers and newborns at birth, and also there was a significant difference between intervention groups and control group in achieving to 80 nmol/L level, 1 month after delivery. Similar to our results, they didn’t observe any adverse events. The significant difference between our study and Hollis et al.’s study is the target group. The method of this study is more powerful in the prevention of vitamin D deficiency adverse effect on pregnancy. 

Also cross sectional studies showed direct relationship between using different doses of vitamin D during pregnancy and vitamin D serum levels (23, 24). In an important retrospective cohort study, Rapuri and Gallagher (25) found that 25(OH) D in case-group women had significantly higher amounts than control-group (P > 0.05). 78% of women, who consumed vitamin D supplement, had appropriate levels of serum 25(OH) D2. All of these findings showed that the long time consumption of vitamin D supplementation (400 IU/day) is an effective method, but low-dosage intake of vitamin D was not an effective way to treat the vitamin D deficiency (23, 26) and scientists suggested higher dosage of supplementation, particularly in reproductive women (20). This study investigated the effect of higher, and safe dosage of vitamin D in the short time of preconception period which is about 2 or 3 month before being pregnant, to avoid concurrency of vitamin D deficiency and the early stages of embryonic life and pregnancy. In regression analysis of the present study, only the basal vitamin D and using vitamin D had a significant correlation with serum vitamin D levels after the intervention (P < 0.001) which is not surprising. The association between vitamin D level and exercise had been shown in elderly population (27), while this study failed to demonstrate the relationship between regular exercise and vitamin D level enhancement among reproductive women.

Confounding factors in achieving normal levels of vitamin D in the study of Larijani et al. (28) which used fortified milk with 600 IU of vitamin D, were the kind of milk and the basal amounts of serum vitamin D (P > 0.001), that the second one is the same as our findings. Factors such as non-Hispanic ethnicity, obesity, smoking, history of diabetes and cardiovascular have been associated with increased risk of vitamin D deficiency and insufficiency. Also supplement consumption, drinking more than one meal milk daily, and exposure to sunlight during the months of May through October have been associated with reduced risk of vitamin D deficiency (P < 0.05) (29). These data were collected from 1814 reproductive women and has high validity due to the large sample size but these findings were not limited to the short time of preconception period. 

We only included women with deficiency because only a few samples had normal baseline level. In this way we would be able to use the results for people with vitamin D deficiency, which constitute very high percentage of our female population. To the authors’ knowledge, these kinds of samples have not been studied exclusively in another trial. Excellent tracking of individuals by the researcher and the loss of only 3 samples and even replacement of them are other strengths of our study. Sampling in the different seasons is the main weakness of this study but the researchers couldn’t be able to modify it. Consumption of vitamin D or calcium during pregnancy could affect serum vitamin D or calcium levels (30, 31), so the impact of 2000 IU/day vitamin D intake during pregnancy on serum vitamin D levels of newborns should be evaluated in future works.

Our data showed that the intake of 2000 IU/day vitamin D for 105 days increased serum levels of this vitamin and brought more than half of women into the normal range. We believe that this therapy in women with high-risk of deficiency, during preconception period is so efficient to prevent the mentioned complications.
